# Invasions of an obligate asexual daphnid species support the nearly neutral theory

**DOI:** 10.1038/s41598-022-11218-4

**Published:** 2022-05-04

**Authors:** Hajime Ohtsuki, Hirotomo Norimatsu, Takashi Makino, Jotaro Urabe

**Affiliations:** Graduate School of Life Sciences, Tohoku University, 6-3 Aoba, Aramaki, Aoba-ku, Sendai, 980-8578 Japan

**Keywords:** Ecology, Evolution

## Abstract

To verify the “nearly neutral theory (NNT),” the ratio of nonsynonymous to synonymous substitutions (*dN/dS*) was compared among populations of different species. To determine the validity of NNT, however, populations that are genetically isolated from each other but share the same selection agents and differ in size should be compared. Genetically different lineages of obligate asexual *Daphnia pulex* invading Japan from North America are an ideal example as they satisfy these prerequisites. Therefore, we analyzed the whole-genome sequences of 18 genotypes, including those of the two independently invaded *D. pulex* lineages (JPN1 and JPN2) and compared the *dN/dS* ratio between the lineages. The base substitution rate of each genotype demonstrated that the JPN1 lineage having a larger distribution range diverged earlier and thus was older than the JPN2 lineage. Comparisons of the genotypes within lineages revealed that changes in *dN/dS* occurred after the divergence and were larger in the younger lineage, JPN2. These results imply that the JPN1 lineage has been more effectively subjected to purification selections, while slightly deteriorating mutations are less purged in JPN2 with smaller population size. Altogether, the lineage-specific difference in the *dN/dS* ratio for the obligate asexual *D. pulex* was well explained by the NNT.

## Introduction

The ratio of nonsynonymous to synonymous substitutions (*dN/dS*) reflects natural selection strength^1–3^. As most nonsynonymous substitutions are deleterious, the *dN/dS* ratio in a population tends to decrease due to negative selection in a given environment. However, if the deleteriousness of the nonsynonymous substitutions is subtle and not immediate, the substitutions would be conserved for a time in small populations that are either recently established or not yet subjected to a strong natural selection. Such possibility was formulated by Ohta^4,5^, who first proposed the nearly neutral theory (NNT), which predicts that the number of nonsynonymous substitutions should be relatively larger in the smaller populations as slightly deleterious mutations can easily spread in the populations by genetic drift. Since then, many studies have provided circumstantial evidence congruent with the NNT^6–12^. For example, some bird species living on islands have more nonsynonymous substitutions in mitochondrial DNA (mtDNA) than their sister species in the mainland^6^. Further, Woolfit and Bromham^7^ found that *dN/dS* ratio is generally higher in insular species than in congeneric mainland species in various taxa, including vertebrates, invertebrates, and plants. As the population sizes of insular species should be smaller than those of congeneric species on continents or the mainland, these phenomena seem to agree well with the expectation of the NNT.

However, these previous studies compared populations of different species in a higher taxonomic group, which may have colonized respective areas at different times following different environmental selections in different habitats. In addition, as different species have evolved to acquire different niches to reduce competition with others and thus utilize different habitats, the difference in the *dN/dS* between the congeneric or sister species may be due to the difference in the ecological factors that regulate their population structures rather than the population size itself^11^. Ideally, to verify the NNT, it is desirable to compare populations of the same species that differ in size and are genetically isolated from each other for long time; this is because these populations likely share the same potential niche, although they may be subjected locally different selective pressures. However, NNT has not yet been tested at the species level.

Panarctic *Daphnia pulex (D.* cf. *pulex* sensu Hebert 1995^13^), a *D. pulex* complex that reside in lakes and ponds, is a native species in North America^14,15^; however, its distribution has expanded to various continents, such as Africa^16^ and Asia^17^, and continental islands, including Japan^18^ and New Zealand^19,20^. This species is originally cyclic parthenogenetic and produces offspring asexually under environmentally favorable conditions, but switches to sexual production to produce resting eggs when environmental conditions are unfavorable. However, some lineages of this species are obligate parthenogenetic and produce asexually resting eggs^21^. Studies suggest that asexual lineages accumulate more deteriorated mutations than sexual lineages because the former cannot purge these mutations due to a lack of meiotic DNA repair^22,23^, although these may not be often passed to the offspring because of strong purifying selections. However, there is growing evidence that the *dN/dS* ratios of asexual lineages are not necessarily greater than those of sexual lineages in the same or congeneric species^10,24–28^, indicating that asexuality is not the sole determinant factor of the *dN/dS* ratio.

So et al.^18^ reported that Panarctic *D. pulex* in Japan are obligate parthenogenetic and are grouped into four distinct lineages, namely JPN1, JPN2, JPN3, and JPN4, based on a partial sequence of the mtDNA. Among these, the JPN1 lineage is distributed throughout the Japanese Islands, while the JPN2 lineage is distributed in the eastern areas of Japan Island. Although JPN3 and JPN4 lineages are limited in both the distribution ranges and the number of genotypes, the JPN1 and JPN2 lineages have several different genotypes^18^. As they are the same species but are genetically isolated from each other due to obligate parthenogenesis, the JPN1 and JPN2 lineages are ideal animals to test the NNT. According to the NNT, the *dN/dS* should be smaller in the JPN1 lineage because this lineage has a larger distribution range and thus, likely a larger population size than the JPN2 lineage^18^.

To test this possibility, we first confirmed the genetic independence of these lineages using the whole-genome of mtDNA and then examined the whole nuclear genomes of *D. pulex* JPN1 and JPN2 lineages to assess their *dN/dS* ratios. As a large part of the difference in *dN* and *dS* among the *D. pulex* lineages likely existed before these lineages diverged from each of their ancestral genotypes, we estimated the number of the nonsynonymous substitution rate against the synonymous substitution rate in the coding regions among the genotypes within each lineage, which served as a gauge to reflect *dN/dS* occurring after the divergence. Then, by comparing the slopes between the JPN1 and JPN2 lineages, we tested the validity of the NNT at the species level.

## Results

**Figure 1:**
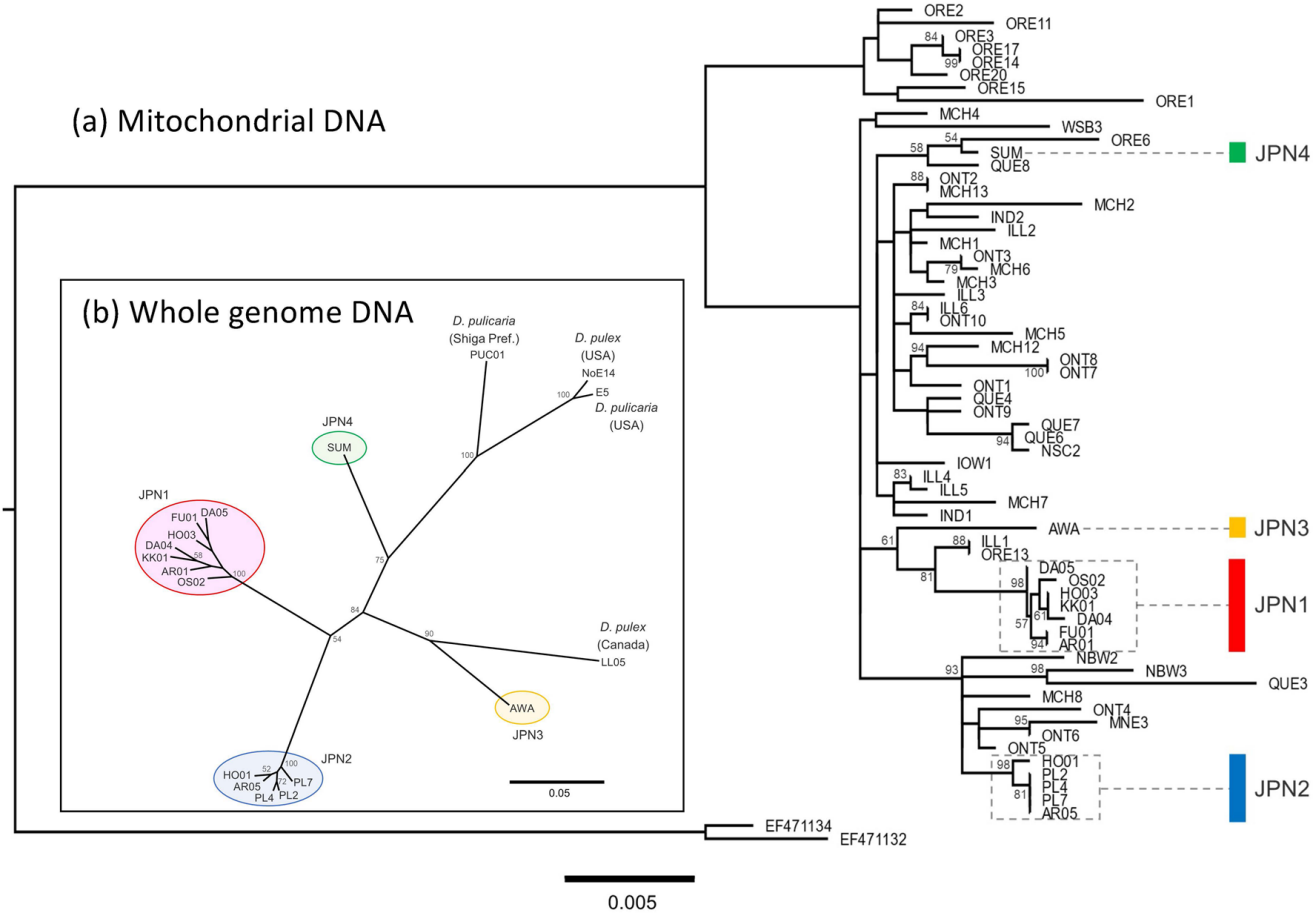
Phylogenetic relationships of the *Daphnia pulex* lineages in Japan. (**a**) Phylogenetic tree of the panarctic *D. pulex* group based on a combined dataset of mitochondrial ND5 and the control region (1747 nucleotide sites including indel). Numbers on branches indicate bootstrap values (> 50 are shown). The analysis included the following samples: seven genotypes in JPN1 (AR01, DA04, DA05, FU01, HO03, KK01, and OS02), five genotypes in JPN2 (AR05, HO01, PL2, P4, and PL7), one genotype in JPN3 (AWA), one genotype in JPN4 (SUM), and genotypes belonging to the North American group described by Paland et al.^21^. Polar *Daphnia pulicaria* (EF471134 and EF471132) were employed as outgroups. (**b**) Phylogenetic tree of *Daphnia pulex* in Japan and other lineages. The tree was constructed based on 5883 SNPs from whole-genome sequence data. Numbers on branches indicate bootstrap values (> 50 are shown). The analysis included the following samples: seven genotypes in JPN1, five genotypes in JPN2, one genotype in JPN3, one genotype in JPN4, and four clones belonging to other lineages besides NoE14, E5, LL05, and PUC01. Species identification was performed using mtDNA.

First, we performed whole mitochondrial genome sequencing of the *Daphnia pulex* genotypes collected in various areas of Japan to assess their genetic relationship. The analysis included specimens reported in previous studies conducted in North America, which is the original distribution range. To construct the phylogenetic tree, we used DNA sequences of ND5 and the control region as the sequences of these regions have been reported for many specimens of *Daphnia pulex* collected in North America^24^ and only few substitutions were found in other regions of the mitochondrial genome. The phylogenetic tree based on mtDNA showed that the genotypes found in Japan were a tiny part of the diversity found in *D. pulex* from North America and that these genotypes were separated into four distinct monophyletic lineages (Fig. 1a), which corresponded to JPN1 to JPN4 identified by So et al.^18^. Among the 21 genotypes in that study, seven and five genotypes were included in the JPN1 and JPN2 lineages, respectively. The number of substitutions in mtDNA examined (15,323 bps), estimated by pairwise comparisons of genotypes, ranged from 0 to 4 and 0 to 11 for JPN1 and JPN2, respectively. Thus, the substitution rate in mtDNA was, on average, 9.32 × 10^–5^ (SD 7.60 × 10^–5^) for JPN1, and 31.3 × 10^–5^ (SD 23.8 × 10^–5^) for JPN2.

Whole-genome DNA sequencing was performed for these genotypes in the JPN1 and JPN2 lineages, with some specimens collected in North America (Table S1). The sequence data of each genotype covered > 135 Mbp of the reference genome data of the *D. pulex* isolate, TCO^29^, according to the BWA software^30^. Using these data, we analyzed phylogenetic relationships among the genotypes. The results confirmed that the JPN1 and JPN2 genotypes constituted different lineages (Fig. 1). In addition, these lineages diverged each from independent ancestral populations. The sequence data were also mapped onto the other reference genome of the *D. pulex* isolate, PA42^31^, which covered > 121 Mbp of this genome data. The number of nuclear DNA substitutions relative to the number of nucleotides examined, estimated by pairwise comparisons of the mapping data onto TCO or PA42 within lineages, were, on average, 1.07 × 10^–5^ (SD 6.75 × 10^–7^) or 1.82 × 10^–5^ (SD 7.44 × 10^–7^) for JPN1, and 0.90 × 10^–5^ (SD 9.30 × 10^–7^) or 1.34 × 10^–5^ (SD 3.20 × 10^–7^) for JPN2 (Table S2). The number was higher in the JPN1 lineage than in the JPN2 lineage using TCO (*t* test: t = 6.435, *p* < 0.001) and PA42 (t = 9.377, *p* < 0.001).

**Figure 2:**
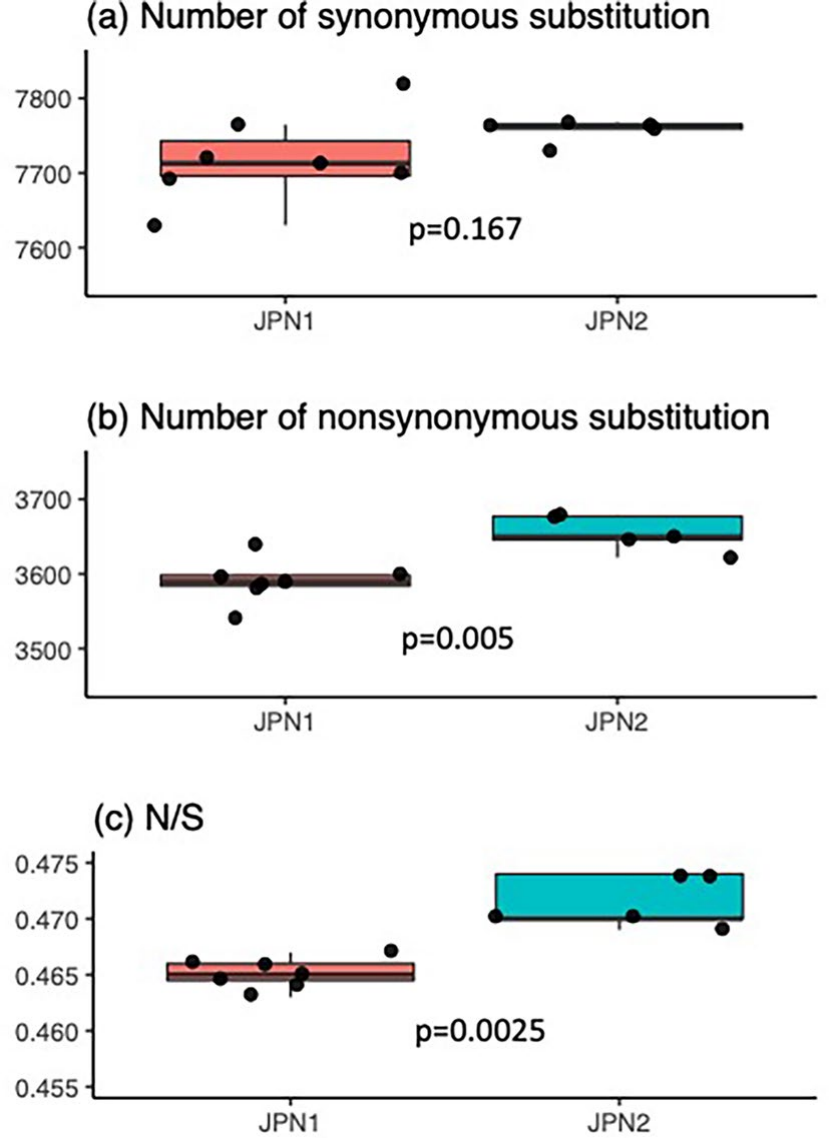
Boxplots showing the numbers of synonymous (**a**) and nonsynonymous substitutions (**b**) and the ratio of these substitutions (**c**) among genotypes of JPN1 and JPN2 lineages. Filled circles show the values of each genotype. The significant probability of difference between the two lineages examined by the Mann–Whitney U test is inserted in each panel.

**Figure 3:**
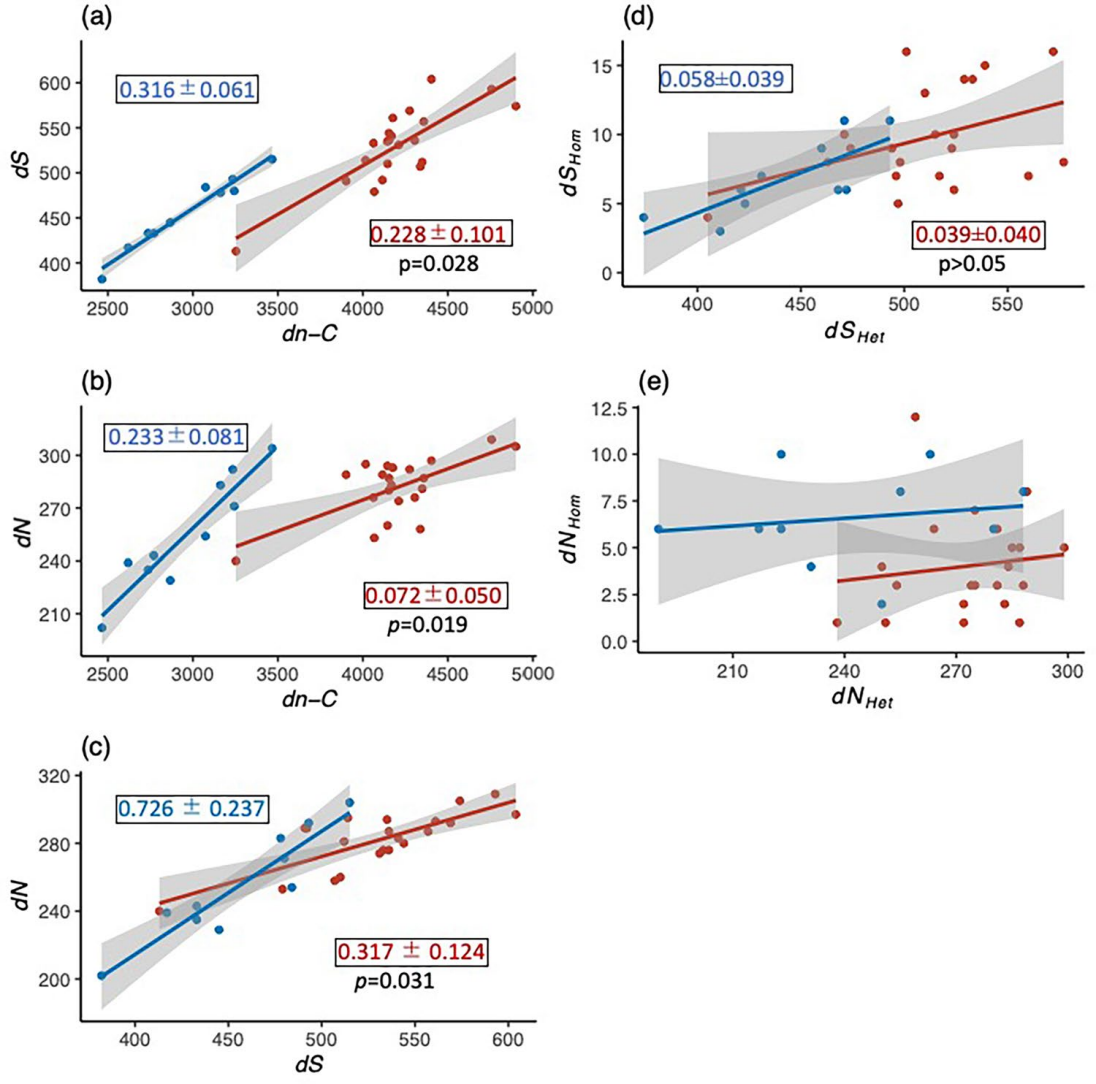
Results of pairwise comparisons among genotypes within lineages for the number of substitutions in the coding and non-coding regions, showing the numbers of synonymous (**a**) and nonsynonymous substitutions plotted against that of substitutions in the non-coding region (**b**), the number of nonsynonymous substitutions plotted against that of synonymous substitutions (**c**), and the number of homozygous substitutions plotted against heterozygous substitutions in synonymous substitutions (**d**) and nonsynonymous substitutions of the coding region (**e**). Red and blue circles indicate JPN1 and JPN2 lineages, respectively. The slopes (mean ± 95%CI) of significant regression lines are shown in squares of each panel with the significant probability (*p*) of difference between the two slopes.

For estimating the numbers of synonymous (*S*) and nonsynonymous substitutions (*N*) for each of the JPN1 and JPN2 genotypes, we used TCO data rather than PA42 as a reference since the former data contained more detailed gene annotation. The results showed that, although *S* did not differ between the JPN1 and JPN2 genotypes (Fig. 2a), *N* was significantly greater in JPN2 than in JPN1 (Fig. 2b). Accordingly, their ratio, *N/S*, was larger in the JPN2 than in the JPN1 lineage (Fig. 2c). It should be noted that the estimates of *S *and *N* above were changeable depending on the data used as reference. Therefore, we estimated the number of substitutions between all pairs of genotypes within lineages (Table S3), which are free from the reference data and occurred after the divergence of the lineages. The numbers of pairwise synonymous substitution (*dS*) and pairwise substitutions in the non-coding region (*dn-C*) differed between JPN1 and JPN2 lineages (*p* < 0.001) but that of pairwise nonsynonymous substitution (*dN*) did not (*p* > 0.05) (Table S3). To examine the difference in ratios between the JPN1 and JPN2 lineages, we plotted the estimations in Fig. 3. The slopes of the regression lines for *dS* and *dN*) plotted against *dn-C* differed significantly between JPN1 and JPN2 (Fig. 3a,b). Moreover, the slope of *dN* against *dS* was significantly greater in JPN2 than in JPN1 (Fig. 3c), indicating that *dN*/*dS* was relatively greater in the former lineage.

Since effects of deleterious mutations likely differ between homozygous and heterozygous substations, by pairwise comparison among genotypes within lineages, we also estimated the number of homozygous and heterozygous substations in both synonymous and nonsynonymous sites without considering the directions from homozygous to heterozygous substitutions and vice versa. The numbers of pairwise homozygous substitutions were also plotted against those of the pairwise heterozygous substitutions for synonymous (*dS*_*Hom*_ vs. *dS*_*Het*_) and nonsynonymous substitutions (*dN*_*Hom*_ vs. *dN*_*Het*_). The slope of *dS*_*Hom*_ against *dS*_*Het*_ did not differ significantly between JPN2 and JPN1 lineages (Fig. 3d). The slope of the regression line for *dN*_*Hom*_ against *dN*_*Het*_ was not significant both in JPN1 (0.023 ± 0.083) and JPN2 lineages (0.014 ± 0.065) (Fig. 3e). However, in the nonsynonymous substitutions, number of the pairwise homozygous substitutions was significantly lower in JPN1 (4.05 ± 1.24) than in JPN2 lineages (6.60 ± 1.79) (*p* < 0.0015) regardless of the pairwise heterozygous substitutions.

For each genotype of the JPN1 and JPN2 lineages, we counted the number of genes with unique (genotype-specific) nonsynonymous substitutions (Table S4). Within the JPN1 lineage, genotype DA04 showed more genes with unique nonsynonymous substitutions (37 genes) than genotype AR01 (22 genes: Table 1). Within the JPN2 lineage, genotype PL7 had much more genes with unique nonsynonymous substitutions (46 genes) than genotype PL2 (18 genes). On average, the number of genes with unique nonsynonymous substitutions was 29.0% for the JPN1 lineage and 32.4% for the JPN2 lineage, with no difference found between the two lineages (*t* test, *p* > 0.1).

**Table 1:** The number of genes with unique nonsynonymous substitutions in each genotype.

Lineage	Genotype	Genes with unique nonsynonymous substitutions
JPN1	AR01	22
	DA04	37
	DA05	31
	FU01	25
	HO03	27
	KK01	31
	OS02	30
JPN2	AR05	39
	HO01	37
	PL2	18
	PL4	22
	PL7	46

## Discussion

The analysis of mitochondrial and nucleic DNA sequences revealed that both the JPN1 and JPN2 lineages of panarctic *D. pulex* (*D*. cf. *pulex,* sensu Hebert 1995^13^) were genetically monophyletic and diverged from different ancestral populations. If the tempo of the substitution rate is the same between the lineages, the JPN1 lineage should be older than JPN2 as the JPN1lineage had a higher number of substitutions in the nucleic DNA among the genotypes than the JPN2 lineage. Based on long-term laboratory experiments over 85 to 170 generations, Keith et al.^32^ estimated a base-substitution rate of 7.17 × 10^–9^ per site per generation for *D. pulex*. Since the generation time of *Daphnia* is on average 10 to 20 days in growing seasons^33,34^, the populations might produce at five generations per year^20^. Based on these numbers, the estimated lineage ages were 508 years old for JPN1 and 372 years for JPN2 based on the data mapped to TCO. The lineage ages based on the data mapped to PA42 were 300 years for JPN1 and 250 years for JPN2. According to Xu et al.^35^, mtDNA substitution rate of asexual *D. pulex* was 4.3 × 10^–8^ mutations per site per generation. If this number and five generations per year were used, the lineage age based on mtDNA was 434 years for JPN1 and 1456 years for JPN2. The estimated age was similar to that based on nuclear DNA in JPN1. However, it was much older than that based on nuclear DNA in JPN2, although the estimated age was somewhat younger than that estimated based on the substitution rate of a partial mtDNA by So et al.^18^. It is well known that molecular dating based on mtDNA is often unreliable, especially when mutation number is limited^36^. Since the number of mutations in mtDNA examined was highly limited in the present genotypes and much smaller than that in nuclear DNA, it is most likely that the substitution rate of mtDNA overestimated the lineage age, especially in JPN2 lineage. Note that number of generations per year may be smaller than five in *D. pulex*, because it is not rare for the population to disappear before summer after producing the resting eggs due to the highly vulnerable to fish predation^37–39^, high water temperature^40,41^, and other environmental stresses^42,43^. Considering this possibility, the lineage age may be older than the above estimates.

*Daphnia* disperses their propagules by producing resting eggs that resist adverse conditions and attach to vector animals that reside in these remote ponds and lakes. Since JPN1 and JPN2 lineages were not monophyletic according to mitochondrial DNA (Fig. 1), it is unlikely that these lineages diverged within Japan. Rather, as panarctic *D. pulex* lineages found in Japanese islands are tiny parts of populations in North America, it is most likely that invasions of this species into Japan occur in very rare events as discussed in So et al.^18^. The possibility implies that different genotypes within a particular lineage invaded Japan independently. One may suspect that several different genotypes of a particular lineage invaded at once. Although this possibility cannot be rejected, it is less likely to have occurred. If this had occurred, several different genotypes of a lineage could be identified within the lakes. However, populations of *D. pulex* lineages in Japanese lakes are generally composed of single genotypes^18^. Thus, it was most probable that these lineages began colonization in Japan from single genotypes and evolved various genotypes.

Since the base substitution number of nuclear DNA was lager in the *D. pulex* JPN1 lineage than JPN2 lineage and since they were obligate parthenogenetic, it is likely that the former lineage immigrated and colonized Japan earlier than the latter lineage, and has the larger effective population size. The inference is congruent with the fact that the distribution range of the JPN1 lineages is larger than that of the JPN2 lineage in the Japanese Islands^18^.

The present study showed that the ratio of synonymous relative to nonsynonymous substitutions in the whole genome differed between the *D. pulex* JPN1 and JPN2 lineages. Specifically, the JPN2 genotypes had more nonsynonymous substitutions than the JPN1 genotypes when these substitutions were counted using the TCO data provided by Colbourne et al.^29^. In both lineages, *N/S* was markedly lower than 1, suggesting that these lineages experienced strong purification selections. However, this may have occurred in ancestral populations before these lineages diverged. To determine how many substitutions occurred after the divergence of these lineages, we carried out pairwise comparisons in the sequence data between pairs of genotypes within lineages and estimated the numbers of synonymous (*dS*) and nonsynonymous substitutions (*dN*) within lineages. Based on the results, the slopes of the nonsynonymous substitution numbers against synonymous substitution numbers were less than one but greater in the JPN2 lineage than in the JPN1 lineage, indicating a higher *dN/dS* ratio in the former lineage. Such findings suggest that JPN1 has been more effectively subjected to purification selection than the JPN2 lineage.

Although, asexual animals tend to accumulate deleterious nonsynonymous mutations because of a lack of recombination^44–46^, several studies showed that the *dN/dS* ratios of asexual genotypes did not differ from those of sexual genotypes in the same animal species^24–28^. Rather, a proposed theory suggests that a large population size can delay the progress of Muller’s ratchet even in asexual organisms^47,48^. This possibility implies that, if asexual animals were greater in the population size in habitats and distributed in larger number of habitats, the *dN/dS* ratio would be lower because they were likely subjected to more purification selections^10^. Supporting this inference, we detected significantly lower *dN/dS* ratios for the *D. pulex* JPN1 lineage that had an earlier divergent time and a larger distribution range than the *D. pulex* JPN2 lineage. Thus, the difference in the *dN/dS* ratio between the two lineages agrees well with the prediction of the NNT. Notably, as observed for asexual oribatid mites^10^, the population size of *Daphnia* is generally very large, with an abundance of 10^3^–10^5^ ind/m^3^, which corresponds to 10^9^–10^11^ individuals in a 10-ha lake with 10-m depth (a moderate size for a lake)^49,50^. If hundreds of lakes were used by *Daphnia* as their habitats, their instantaneous abundance would reach 10^11^–10^13^ individuals; this number might be modest. Accordingly, asexual *D. pulex* lineages in Japan may be able to escape mutation meltdown for a long time.

Tucker et al.^28^ suggested that evolutional longevity of asexual *D. pulex* should be short because of the loss of heterozygosity due to gene conversion or base mutations that expose recessive deleterious alleles. If this is the case, genotypes of a long-lasting lineage in nature should have a lower ratio of homozygous relative to heterozygous because genotypes homozygous for recessive deleterious alleles are quickly purged. Supporting this inference, the number of pairwise homozygous substations relative to the pairwise heterozygous substitutions in nonsynonymous mutations was significantly lower among genotypes in old JPN1 lineage than those among younger JPN2 lineage. The result again accords well with the expectation from NNT.

One may suspect that the difference in the *dN/dS* ratio between *D. pulex* JPN1 and JPN2 lineages was caused by a large difference in selective agents. As shown in the present study, non-synonymous substitutions occurred at different positions in the genome among the genotypes in both the JPN1 and JPN2 lineages (Tables 1 and S4), suggesting that each genotype evolved genetically unique traits (phenotype). According to a priority theory, an early arrival population can monopolize newly habitats if they have enough time to adapt to these habitats before the invasion of subsequent populations^51,52^. A recent study showed that *D. pulex* has a relatively larger proportion of duplicated genes in their genome than other animals^53^. As duplicated genes are a major source of adaptability^54^, this species may have the ability to produce various genotypes that can colonize new habitats without recombination. If JPN1 genotypes established their populations and occupied most of the standing niches in Japanese lakes through such challenges, novel traits fixed by point mutations might have been more important for JPN2 lineages to exploit new habitats or vacant niche spaces. Accordingly, the JPN2 genotypes may have higher *dN/dS* ratios than the JPN1 genotypes. However, this possibility cannot explain why such positive selection or relaxation of negative selection did not occur within the JPN1 lineage.

As the JPN2 lineage was a latecomer and had a smaller effective population size, it is highly probable that they had not yet been subjected to purifying selections for a long time, like the JPN1 lineage. This scenario is in accordance with the findings in the long-term evolution experiment of bacteria with large population sizes where various mutants were accumulated at the onset of evolution due to relaxed selection (high *dN/dS* ratio) but were eventually less accumulated due to negative selections caused by the increased competitive interactions among the indigenous mutants, resulting in a decrease in the *dN/dS* ratio over time (^55^, see also review by Rocha^56^). In the case of panarctic *D. pulex*, the JPN1 genotypes could survive in the environmental conditions of Japanese lakes due to a strong negative selection generated after the genetic divergence within lineages.

## Conclusion

Although many studies have presented evidence congruent with NNT by comparing the genome among different species, few studies have examined the validity of the NNT at the species level. By examining genetically different lineages within panarctic asexual *D. pulex*, the present study showed that the lineage diverged at an earlier time and occupied a larger distribution range had a lower *dN/dS* ratio, indicating that most phenotypically related mutations were not advantageous for increasing fitness but rather deteriorated under given habitats. Thus, the relatively higher *dN/dS* ratio in the younger lineage, whose distribution range was not yet expanded, implies that nearly deteriorating mutations have yet to be purged. The lineage-specific difference in the *dN/dS* ratio of panarctic *D. pulex* in Japan indicates that the "NNT" by Ohta^4,5^ plays a fundamental role in the evolution of this species.

## Methods

### Study populations

The locations of lakes where genotypes of panarctic *D. pulex* were collected are shown in Table S1. We identified species of the genotypes according to the 12S mtDNA described by So et al.^18^. To reconstruct the phylogenetic relationships among these genotypes, we used a genotype of *D. pulricaria* collected in Japan and the genotypes of these species collected in North America as out groups (Table S1). These genotypes were cultured for more than 5 years in the authors’ institution laboratory under constant environmental conditions.

### Mitochondrial DNA sequences

A single individual of each genotype was used to determine the sequences of mtDNA. The DNA extraction procedure for sequencing is described elsewhere^18^. Extracted DNA was amplified by PCR using three primer sets designed to cover the whole mitochondrial genome of panarctic *D. pulex* (Table S5). The 20-µl mix for each reaction consisted of 1.5 µL of extracted DNA, 0.4 units of KOD FX Neo (TOYOBO), 10 µL of KOD FX Neo buffer, 4.0 µL of each 2.0 mM dNTP, and 0.2 µM of each primer. The thermal cycling conditions were as follows: a 2 min initial cycle at 94 °C, followed by 30 cycles of 98 °C for 10 s and 68 °C for 3 min using primer set 1 (Dpu_06487F1 and Dpu_14188R1); a 2 min initial cycle at 94 °C, followed by 30 cycles of 98 °C for 10 s, 65 °C for 30 s, and 68 °C for 4 min using set 2 (Dpu_01488F2 and Dpu_06899R2); and a 2 min initial cycle at 94 °C, followed by 30 cycles of 98 °C for 10 s, 64 °C for 30 s, and 68 °C for 2 min using set 3 (Dpu_13944F3 and Dpu_02003R3). Each amplified product was sequenced by primer walking using newly designed specific primers. The products were purified by ExoSAP-IT^(R)^ (Affymetrix) and sequenced using a Big-Dye™ Terminator v3.1 Cycle Sequencing Ready Reaction Kit (Thermo Fisher Scientific), according to the method described by So et al.^18^. All primers used for sequencing are listed in Table S5. The sequencing reactions were purified using a BigDye XTerminator^(R)^ Purification Kit (Thermo Fisher Scientific) and analyzed using an ABI PRISM^(R)^ 3100-Avant Genetic Analyzer. All sequencing data were deposited in DDBJ under accession numbers, LC632382 to LC632395 (Table S1).

Using the sequences in this study and genotypes belonging to the panarctic group^21^, a phylogenetic tree based on ND5 and the control region was constructed via maximum likelihood (ML) analysis. The sequences were aligned using MAFFT v7.475^57^ and then visually checked and edited. A model with the lowest corrected Akaike information criterion (AICc) by model selection using Kakusan4^58^ was selected as the best model. ML analysis with 100 bootstrap replicates was performed using RAxML^59^ with the GTRGAMMA model.

### Nuclear genome sequencing

Whole-genome sequencing was performed according to a previous study^60^. Fifty to seventy individuals of each genotype were collected for DNA extraction using a Maxwell^(R)^ 16 instrument (Promega, Madison, WI, USA) and Maxwell^(R)^ 16 LEV Plant DNA Kit (Promega). Construction and sequencing libraries were performed at the Beijing Genomics Institute (BGI Japan; Kobe, Japan). The libraries were constructed using a unique method developed by BGI JAPAN (low input method) from more than 500 ng of DNA per sample. Sequencing was conducted on the Illumina Hiseq X™ Ten platform (Illumina, San Diego, CA, USA) with a paired-end 150 bp (PE150) strategy to obtain approximately 8 Gb of data per sample (approximately 40 × coverage). The data were filtered using SOAPunke software^61^ with the following options: -n 0.1, -l 10, -q 0.5, -i, and -A 0.5. Reads of the individual FASTQ files were mapped to the reference genome data of the *D. pulex* isolates, TCO^29^ and PA42^31^, using BWA^30^ with mem command and the following options: -M, -A 1, -B 40, -O 10, and -E 3. Removal of potential PCR duplicates and detection of polymorphisms in the data were conducted using SAM tools^62^. Sequencing data with a > 20 quality score were used for subsequent analyses. The raw sequencing data of the genotypes in this study have been deposited in the DDBJ Sequence Read Archive under the accession numbers, SAMD00322344 to SAMD00322361 (Table S1).

### Phylogenetic analysis

An unrooted phylogenetic tree was constructed by the maximum likelihood (ML) method based on the SNP data to clarify the phylogenetic relationship among genotypes. SNPhylo pipeline^63^ was used for this analysis with the options: -a 15,311, -b 100, and -H.

### Analysis of DNA substitutions

The number of substitutions in each genotype was calculated in two ways: the number of substitutions estimated in comparison with the reference data and pairwise comparisons between genotypes within each lineage. In this calculation, we removed all gaps in whole-genome alignments and used sequences that were common to all the genotypes examined. Using the data of each genotype mapped to TCO^29^ or PA42^31^, we estimated the proportion of substitutions to the whole genome as a substitution rate (*N*_*sub*_) as follows:

$$N_{sub} = \, \left( {2hom + het} \right)/\left( {{\text{the}}\;{\text{number}}\;{\text{of}}\;{\text{nucleotide}}\;{\text{examined}}} \right),$$

where *hom* and *het* are the number of sites where both of the nucleotides were substituted relative to reference genome (homozygous substitutions) and those where one of the nucleotides was substituted (heterozygous substitutions), respectively.

The numbers of synonymous (*S*) and nonsynonymous (*N*) substitutions in the amino-acid coding regions were also estimated for each genotype of the JPN1 and JPN2 lineages by comparing the sequence data with TCO or PA42. The ratios of synonymous (*S*) and nonsynonymous substitutions (*N*) in the coding regions were calculated for each genotype. Statistical difference in the estimates between the lineages was examined by the Mann–Whitney *U*-test with Bonferroni corrections.

Using the pairwise comparison of the sequence data between genotypes within each lineage, we estimated the number of substitutions in the non-coding regions (*dn-C*) and synonymous (*dS*) and nonsynonymous substitutions (*dN*) in the coding regions, because neutrality differed among the substitutions in these regions. The coverage for each substitution was at least five, generally > 10. Then, we plotted the numbers of *dN* or *dS* against that of *dn-C*, the number of *dN* against that of *dS*, and estimated the slopes between these two variables using conventional regression analysis. Additionally, for each pair of genotypes within the same lineage, the nonsynonymous and synonymous substitutions in the coding region were sorted into heterozygous (*dN*_*Het*_ and *dS*_*Het*_) or homozygous substitutions (*dN*_*Hom*_ and *dS*_*Hom*_). Then, in each of the JPN1 and JPN2 lineages, we plotted the number of *dN*_*Hom*_ (or *dS*_*Hom*_) against *dN*_*Het*_ (or *dS*_*Het*_) in the same way above. Finally, the statistical differences in the estimated slopes between the JPN1 and JPN2 lineages were examined by a randomization test. In this test, we first estimated the difference in the observed slopes of the regression lines between JPN1 and JPN2 lineages. For the randomization test, we pooled data of JPN1 and JPN2 lineages. Then, in each of the JPN 1 and JPN2 lineages, we randomly selected the same numbers of data to the original samples from the pooled data allowing replacement. Using these randomization data, we performed the regression analysis for estimating the slope and calculated the difference in the slopes between JPN1 and JPN2 lineages. We repeated this procedure 1999 times. We concluded that the slope across genotypes differed significantly between JPN1 and JPN2 if the observed difference in the slope was larger than 95% of the difference in the slopes estimated by the randomization procedure. In the analysis of *dN*_*Hom*_ against *dN*_*Het*_, the regression line was not significant both for JPN1 and JPN2 linages. In this case, we estimated the difference in *dN*_*Hom*_ between the two lineages without considering the explanatory effects of *dN*_*Het*_. Then, a significant difference in the observed difference was examined by the randomization procedure as above. These analyses were performed using the built-in package of R version 3.6.1^64^.

## Supplementary Information

Supplementary Information.

## Data Availability

DNA sequence data analyzed in this study are depoßsited in the GenBank, and accession numbers are listed in Table S1 of supplemental information. Data used in Figs. 2 and 3 are deposited in Dryad (https://doi.org/10.5061/dryad.n5tb2rbx6).
