# Supplementary material for: Invasions of an obligate asexual daphnid species support the nearly neutral theory
Source: Sci Rep. 2022 May 4;12:7305. doi: 10.1038/s41598-022-11218-4 (PMC9068809; doi:10.1038/s41598-022-11218-4)
Supplement: Supplementary file 1 — Supplementary Information. [file 41598_2022_11218_MOESM1_ESM.pdf]

Supporting information Table S1-S5

Invasions of an obligate asexual daphnid species support the nearly neutral theory

Hajime Ohtsuki[1], Hirotomo Norimatsu[1], Takashi Makino[1] and Jotaro Urabe[1*]

Affiliations

[1]Graduate School of Life Sciences, Tohoku University, 6-3 Aoba, Aramaki, Aoba-ku, Sendai, 980-8578, Japan

* Corresponding author: Jotaro Urabe (urabe@tohoku.ac.jp)

Table S1. Information on *Daphnia pulex* specimens analyzed in the present study.

Species	Lineage	Genotype	Lake	Area	Nation	Latitude (°N)	Longitude (°E)	DDBJ Accesion number	
								Whole nuclear genome	Whole mitocondorial genome
D. pulex	JPN1	AR01	Arigatani Ike	Shizuoka	Japan	34.691	138.126	SAMD00322344	LC632382
D. pulex	JPN1	DA04	Daizahoshi Ike	Nagano	Japan	36.706	138.145	SAMD00322345	LC632383
D. pulex	JPN1	DA05	Daizahoshi Ike	Nagano	Japan	36.706	138.145	SAMD00322346	LC632384
D. pulex	JPN1	FU01	Furuichi Oike	Tottori	Japan	35.391	133.339	SAMD00322347	LC632385
D. pulex	JPN1	HO03	Hataya Onuma	Yamagata	Japan	38.245	140.204	SAMD00322348	LC632386
D. pulex	JPN1	KK01	Kokenuma	Yamagata	Japan	38.231	140.195	SAMD00322349	LC632387
D. pulex	JPN1	OS02	Osawa Tameike	Miyagi	Japan	38.439	140.919	SAMD00322350	LC632388
D. pulex	JPN2	AR05	Arigatani Ike	Shizuoka	Japan	34.691	138.126	SAMD00322351	LC632389
D. pulex	JPN2	HO01	Hataya Onuma	Yamagata	Japan	38.245	140.204	SAMD00322352	LC632390
D. pulex	JPN2	PL2	Fukami Ike	Nagano	Japan	35.326	137.821	SAMD00322353	LC632391
D. pulex	JPN2	PL4	Fukami Ike	Nagano	Japan	35.326	137.821	SAMD00322354	LC632392
D. pulex	JPN2	PL7	Fukami Ike	Nagano	Japan	35.326	137.821	SAMD00322355	LC632393
D. pulex	JPN3	AWA	Awaji Nariai-ji	Hyogo	Japan	34.283	134.809	SAMD00322356	LC632394
D. pulex	JPN4	SUM	Sumiyoshi Ike	Kagoshima	Japan	31.772	130.592	SAMD00322357	LC632395
D. pulicaria		PUC01	Lake Biwa	Shiga	Japan	35.191	135.994	SAMD00322358	-
D. pulicaria		E5	a small lake	NY	USA			SAMD00322359	-
D. pulex		NoE14	a small lake	NY	USA			SAMD00322360	-
D. pulex		LL05	a small lake	Manitoba	Canada			SAMD00322361	-

Table S2. Substitution rates and estimated divergence times for each lineage

Lineage	Reference	Avg. subst. rate	Div.time (3gen/yr)	Div.time (5en/yr)	Div.time (10gen/yr)
JPN1	TCO	1.07E-05	848	509	254
	PA42	1.82E-05	499	299	150
JPN2	TCO	9.00E-06	621	373	186
	PA42	1.34E-05	416	250	125

Table S3. The numbers of nonsynonymous and synonymous substitutions and that in non-coding regions estimated by the pairwise comparisons between genotypes within JPN1 and JPN2 lineages, and results of t-test examining the difference between these lineages

Lineage	Pair	Nonsynonymous	Synonymous	Non-coding
		(*ΔNsy*)	(*ΔSyn*)	(*Δnon-C*)
JPN1	AR01vsDA04	292	569	4275
	AR01vsDA05	309	593	4757
	AR01vsFU01	305	574	4900
	AR01vsHO03	283	541	4168
	AR01vsKK01	297	604	4403
	AR01vsOS02	280	544	4153
	DA04vsDA05	295	514	4016
	DA04vsFU01	289	491	3901
	DA04vsHO03	289	492	4115
	DA04vsKK01	293	561	4176
	DA04vsOS02	276	533	4062
	DA05vsFU01	240	413	3253
	DA05vsHO03	276	536	4305
	DA05vsKK01	294	535	4144
	DA05vsOS02	287	557	4358
	FU01vsHO03	258	507	4338
	FU01vsKK01	260	510	4145
	FU01vsOS02	281	512	4349
	HO03vsKK01	274	531	4211
	HO03vsOS02	253	479	4067
	KK01vsOS02	287	536	4154
JPN2	AR05vsHO01	254	484	3074
	AR05vsPL2	229	445	2867
	AR05vsPL4	243	433	2770
	AR05vsPL7	292	493	3234
	HO01vsPL2	239	417	2619
	HO01vsPL4	235	433	2736
	HO01vsPL7	304	515	3466
	PL2vsPL4	202	382	2465
	PL2vsPL7	271	480	3243
	PL4vsPL7	283	478	3162
t-test exaining statistical difference begtween JPN1 and JPN2 lineages				
t -value		3.582	6.445	29.540
p -value		> 0.05	< 0.001	< 0.001

Table S4. List of genes with unique nonsynonymous substitutions in each genotype.

Data are listed in the order corresponding to gene stable IDs of TCO (Colbourne et al. 2011 [29]). Filled and open circles denote homozygous and heterozygous mutations on the gene, respectively.

	JPN1							JPN2				
Gene stable ID	AR01	DA04	DA05	FU01	HO03	KK01	OS02	AR05	HO01	PL2	PL4	PL7
DAPPUDRAFT_100142										○		
DAPPUDRAFT_100184											○	
DAPPUDRAFT_100200											●	
DAPPUDRAFT_101046						○						
DAPPUDRAFT_101087									○			
DAPPUDRAFT_102050	●											
DAPPUDRAFT_102106									○			
DAPPUDRAFT_102133	●											
DAPPUDRAFT_102173												●
DAPPUDRAFT_102448												●
DAPPUDRAFT_102589						○						
DAPPUDRAFT_102606							●					
DAPPUDRAFT_102698	○											
DAPPUDRAFT_103010					○							
DAPPUDRAFT_103833				●								
DAPPUDRAFT_104592								○				
DAPPUDRAFT_104787	○											
DAPPUDRAFT_104885						○						
DAPPUDRAFT_105173			○								○	
DAPPUDRAFT_105768								○				
DAPPUDRAFT_106149	○											
DAPPUDRAFT_106477					●							
DAPPUDRAFT_106666					●							
DAPPUDRAFT_106883								○				
DAPPUDRAFT_106899		○							●			

DAPPUDRAFT_107340		○										
DAPPUDRAFT_107700					●							
DAPPUDRAFT_108150											○	
DAPPUDRAFT_108326		○										
DAPPUDRAFT_108478								○				
DAPPUDRAFT_109031								○				
DAPPUDRAFT_109282		●										
DAPPUDRAFT_109456			○									○
DAPPUDRAFT_109764				○								
DAPPUDRAFT_109956								○				
DAPPUDRAFT_110077										○		
DAPPUDRAFT_110200							○					
DAPPUDRAFT_110341											○	
DAPPUDRAFT_110847									○			
DAPPUDRAFT_111210									○			
DAPPUDRAFT_111549									●			
DAPPUDRAFT_113018										○		
DAPPUDRAFT_113547									●			
DAPPUDRAFT_113947										○		
DAPPUDRAFT_114229	●											
DAPPUDRAFT_114231				○								
DAPPUDRAFT_114273											●	
DAPPUDRAFT_114320						○						
DAPPUDRAFT_114421							○					
DAPPUDRAFT_114604												○
DAPPUDRAFT_114926									○			
DAPPUDRAFT_116883							●					
DAPPUDRAFT_116884												●
DAPPUDRAFT_117019											●	
DAPPUDRAFT_117545			○									
DAPPUDRAFT_117736								○				
DAPPUDRAFT_117737			○									
DAPPUDRAFT_117879							○					○
DAPPUDRAFT_119275										●		
DAPPUDRAFT_119531								○				

DAPPUDRAFT_120478			○									
DAPPUDRAFT_120630									○			
DAPPUDRAFT_123888								●				
DAPPUDRAFT_124162	○											
DAPPUDRAFT_127260										○		
DAPPUDRAFT_1338									○			
DAPPUDRAFT_186887								○				
DAPPUDRAFT_188071			○									
DAPPUDRAFT_194168					●							
DAPPUDRAFT_194355					○							
DAPPUDRAFT_196016							○					
DAPPUDRAFT_197829												●
DAPPUDRAFT_199479						○						
DAPPUDRAFT_200371					○							
DAPPUDRAFT_200491						○						
DAPPUDRAFT_201971						○						
DAPPUDRAFT_204745					●							
DAPPUDRAFT_208272		○										
DAPPUDRAFT_210457									○			
DAPPUDRAFT_213335						○						
DAPPUDRAFT_214350		○										
DAPPUDRAFT_218706								○				
DAPPUDRAFT_218819								○				
DAPPUDRAFT_220911										○		
DAPPUDRAFT_221352									○			
DAPPUDRAFT_221821				●								
DAPPUDRAFT_222068					●							
DAPPUDRAFT_222344						○						
DAPPUDRAFT_223107									○			
DAPPUDRAFT_223110	○											
DAPPUDRAFT_224080							●					
DAPPUDRAFT_226467								○				
DAPPUDRAFT_227934			●	○								
DAPPUDRAFT_228516			○									
DAPPUDRAFT_228726		○										

DAPPUDRAFT_231325					●							
DAPPUDRAFT_231992							○					
DAPPUDRAFT_234464		○										
DAPPUDRAFT_234592	○											
DAPPUDRAFT_235227								○				
DAPPUDRAFT_235623												○
DAPPUDRAFT_235675								●				
DAPPUDRAFT_235814												●
DAPPUDRAFT_236078												○
DAPPUDRAFT_236114		○										
DAPPUDRAFT_236160						○						
DAPPUDRAFT_237497			○									
DAPPUDRAFT_237515							○				●	
DAPPUDRAFT_238573												●
DAPPUDRAFT_238928			○									
DAPPUDRAFT_238989								●				
DAPPUDRAFT_239551					○							
DAPPUDRAFT_239781			○					○				
DAPPUDRAFT_239901							○					
DAPPUDRAFT_240350									●			
DAPPUDRAFT_240561									○			
DAPPUDRAFT_240676								○				
DAPPUDRAFT_241840					●							
DAPPUDRAFT_242342		○										
DAPPUDRAFT_242810	○											
DAPPUDRAFT_244506	○											
DAPPUDRAFT_244635									○			
DAPPUDRAFT_246189	●											
DAPPUDRAFT_246435												
DAPPUDRAFT_246486												○
DAPPUDRAFT_247464	○											
DAPPUDRAFT_247774								●				
DAPPUDRAFT_248360				○								
DAPPUDRAFT_249918											○	
DAPPUDRAFT_250461												○

DAPPUDRAFT_251296									●			
DAPPUDRAFT_251607			○							○		
DAPPUDRAFT_251710	●											
DAPPUDRAFT_251767						○						
DAPPUDRAFT_253359				●								
DAPPUDRAFT_253382		●										
DAPPUDRAFT_253578			○									
DAPPUDRAFT_253899										○		
DAPPUDRAFT_254022						●						
DAPPUDRAFT_254610					●							
DAPPUDRAFT_256362												○
DAPPUDRAFT_258674	●					○						
DAPPUDRAFT_259829										○		
DAPPUDRAFT_259872				○								
DAPPUDRAFT_260043									○			
DAPPUDRAFT_260396										○		
DAPPUDRAFT_260662			○									
DAPPUDRAFT_262014		○										
DAPPUDRAFT_262510		○										
DAPPUDRAFT_263269				○								
DAPPUDRAFT_263370				●								
DAPPUDRAFT_263616						○					○	
DAPPUDRAFT_263869								○				
DAPPUDRAFT_264458					○							
DAPPUDRAFT_264791											○	
DAPPUDRAFT_264813							●					
DAPPUDRAFT_265215									○			
DAPPUDRAFT_265372		●							○			
DAPPUDRAFT_265628	○											
DAPPUDRAFT_265983												○
DAPPUDRAFT_266275											●	
DAPPUDRAFT_266638			○									○
DAPPUDRAFT_271148												○
DAPPUDRAFT_299651												○
DAPPUDRAFT_299927			○									

DAPPUDRAFT_300404												●
DAPPUDRAFT_300508									○			
DAPPUDRAFT_301167								○				
DAPPUDRAFT_301201			○									
DAPPUDRAFT_301407					○							
DAPPUDRAFT_301532												○
DAPPUDRAFT_301877							●					
DAPPUDRAFT_301897						○						
DAPPUDRAFT_302117									○			
DAPPUDRAFT_302134							○					
DAPPUDRAFT_302138						○						
DAPPUDRAFT_302484		●										
DAPPUDRAFT_302612					○							
DAPPUDRAFT_302657						○						
DAPPUDRAFT_302833												
DAPPUDRAFT_302844								●				
DAPPUDRAFT_302885										○		
DAPPUDRAFT_303105											○	
DAPPUDRAFT_303198		●										●
DAPPUDRAFT_303205			○									
DAPPUDRAFT_303246						○				●		
DAPPUDRAFT_303405					○							
DAPPUDRAFT_303483												○
DAPPUDRAFT_303885									○			
DAPPUDRAFT_303904									○			
DAPPUDRAFT_303927	○											
DAPPUDRAFT_304023												●
DAPPUDRAFT_304124	○								○			
DAPPUDRAFT_304125									○			
DAPPUDRAFT_304131					○							
DAPPUDRAFT_304382									○			
DAPPUDRAFT_304684											○	
DAPPUDRAFT_304801				○								
DAPPUDRAFT_304875												
DAPPUDRAFT_304939						○						

DAPPUDRAFT_304943				●							○	
DAPPUDRAFT_305286				○								
DAPPUDRAFT_30529				○								
DAPPUDRAFT_305332						○						
DAPPUDRAFT_305389								○				
DAPPUDRAFT_305668			○									
DAPPUDRAFT_305909			○									○
DAPPUDRAFT_306244												○
DAPPUDRAFT_306421										○		
DAPPUDRAFT_30674							○					
DAPPUDRAFT_306779							○	○				
DAPPUDRAFT_306814								●				
DAPPUDRAFT_306924									○			
DAPPUDRAFT_307384				○								
DAPPUDRAFT_307822												○
DAPPUDRAFT_307855	○											
DAPPUDRAFT_308050												
DAPPUDRAFT_308560	○											
DAPPUDRAFT_308709							○					
DAPPUDRAFT_308845						○						
DAPPUDRAFT_308901					○							
DAPPUDRAFT_30917									○			
DAPPUDRAFT_309233												○
DAPPUDRAFT_309396											○	
DAPPUDRAFT_309401						○						
DAPPUDRAFT_309961					●							
DAPPUDRAFT_310325			○									
DAPPUDRAFT_310536							○					
DAPPUDRAFT_310578							○					
DAPPUDRAFT_311177							○					
DAPPUDRAFT_311292								○				
DAPPUDRAFT_311786		●										
DAPPUDRAFT_311898			○									
DAPPUDRAFT_312544			○									
DAPPUDRAFT_313288	○											

DAPPUDRAFT_313504												○
DAPPUDRAFT_313554		●										
DAPPUDRAFT_314003					○							
DAPPUDRAFT_314497					●							
DAPPUDRAFT_314878								○				
DAPPUDRAFT_315894						○						
DAPPUDRAFT_316279			○									
DAPPUDRAFT_316320		○										
DAPPUDRAFT_316647												
DAPPUDRAFT_317276						○						
DAPPUDRAFT_317513				○								
DAPPUDRAFT_318035			○									
DAPPUDRAFT_318220		○										
DAPPUDRAFT_318416								○				
DAPPUDRAFT_318862									○			
DAPPUDRAFT_319139									○			
DAPPUDRAFT_319198												○
DAPPUDRAFT_319275		○										
DAPPUDRAFT_319459			○	●								
DAPPUDRAFT_319496						○						
DAPPUDRAFT_319819				●				●				
DAPPUDRAFT_319936				○								
DAPPUDRAFT_320142								○				
DAPPUDRAFT_320244									○			
DAPPUDRAFT_321105						●						
DAPPUDRAFT_322033		○										○
DAPPUDRAFT_322293									○			
DAPPUDRAFT_323470		○										
DAPPUDRAFT_323689						○						
DAPPUDRAFT_323811												○
DAPPUDRAFT_324583												○
DAPPUDRAFT_324745												○
DAPPUDRAFT_325092						○						
DAPPUDRAFT_325573			○									
DAPPUDRAFT_325621			○									

DAPPUDRAFT_325795							○					
DAPPUDRAFT_325898					●							
DAPPUDRAFT_326717												○
DAPPUDRAFT_326941			○									
DAPPUDRAFT_326957									○			
DAPPUDRAFT_327022											○	
DAPPUDRAFT_327679								●				
DAPPUDRAFT_328037		○										
DAPPUDRAFT_328318							○					
DAPPUDRAFT_328712				●								
DAPPUDRAFT_329106			○									
DAPPUDRAFT_329385								○				
DAPPUDRAFT_329664							○					○
DAPPUDRAFT_329805						●						
DAPPUDRAFT_329962											○	
DAPPUDRAFT_330286											●	
DAPPUDRAFT_330570					○							
DAPPUDRAFT_330625												●
DAPPUDRAFT_330844								○				
DAPPUDRAFT_330970											○	
DAPPUDRAFT_331526												○
DAPPUDRAFT_331838					●							
DAPPUDRAFT_332703					○		○					
DAPPUDRAFT_332853							○					○
DAPPUDRAFT_33293					○							
DAPPUDRAFT_332948		○										
DAPPUDRAFT_333387				○								
DAPPUDRAFT_333581										○		
DAPPUDRAFT_333653							○					
DAPPUDRAFT_333828				○								
DAPPUDRAFT_334025							○					
DAPPUDRAFT_334105				○								
DAPPUDRAFT_334794		●										
DAPPUDRAFT_335346										○		
DAPPUDRAFT_335601							●					

DAPPUDRAFT_335789		○										
DAPPUDRAFT_340525												○
DAPPUDRAFT_340854								●				
DAPPUDRAFT_346846		○										
DAPPUDRAFT_346847								●				
DAPPUDRAFT_347258								○				●
DAPPUDRAFT_347585		●										
DAPPUDRAFT_347609				○								
DAPPUDRAFT_40007	●											
DAPPUDRAFT_40651												○
DAPPUDRAFT_41180					●							
DAPPUDRAFT_41292						○						
DAPPUDRAFT_42306										●		
DAPPUDRAFT_42461		●							●			
DAPPUDRAFT_442477						○						
DAPPUDRAFT_45783				●								
DAPPUDRAFT_47834			○						○			
DAPPUDRAFT_52115									○			○
DAPPUDRAFT_52511			○									
DAPPUDRAFT_55188												○
DAPPUDRAFT_58148								○				
DAPPUDRAFT_58385												●
DAPPUDRAFT_58979							○					
DAPPUDRAFT_59583												○
DAPPUDRAFT_59739			○									
DAPPUDRAFT_60562							○					
DAPPUDRAFT_60927												●
DAPPUDRAFT_62213								○				
DAPPUDRAFT_64710	○											
DAPPUDRAFT_65729		●										
DAPPUDRAFT_7338		○										
DAPPUDRAFT_93942		○										
DAPPUDRAFT_94669				○								
DAPPUDRAFT_94746									○			
DAPPUDRAFT_95082											○	

DAPPUDRAFT_97099												○
DAPPUDRAFT_97244							○					
DAPPUDRAFT_97326								○				
DAPPUDRAFT_97672							○					
DAPPUDRAFT_98367						○						
DAPPUDRAFT_99493				○								
DAPPUDRAFT_99701								●				
DAPPUDRAFT_99799											○	

Table S5. List of primers for sequencing whole genome DNA. Names of primer used for amplifying each region (set 1, 2 and 3) are shown in bold.

	name	sequence
Set 1	**Dpu_01488F1**	GCTCTTAGACTTTTAATTCGCGCCG
	Dpu_02163F1	ATTATTAGCCACGAAAGAGG
	Dpu_02863F1	CTTCAATCGAATGACAGCATTC
	Dpu_03521F1	ACGCCGTACCTGGTCGTTTAAATC
	Dpu_04918R1	GAGGCAATGAACAAGATTATTCCC
	Dpu_05556R1	CAAGACCACAGATCTAATTGTC
	Dpu_06200R1	TCAATGATTTGAGAACAGCT
	**Dpu_06899R1**	GCCTTTCTTAGCGGGGTTTTACTCT
Set 2	**Dpu_06487F2**	AACCTCTAAACTTCCTAACTGCCCC
	Dpu_07124F2	TCTAAGGGTAGACAATGCAA
	Dpu_07650F2	GATAGCCATAGAAGCAACAA
	Dpu_07822F2	TTCTCCCCCCTAAAAATCACTC
	Dpu_08126F2	CTGTCTGTTCAATGTTGGGTAGAC
	Dpu_08436R2	ATTAGTCACGGCCTCTGTTC
	Dpu_08641F2	AGGGACAACTCGAACTATTCCG
	Dpu_09209F2	AGGCCTAATCCAAACCCGCTTA
	Dpu_09729F2	ACACCCAAAGCTCCCAAAGCTCCA
	Dpu_10758R2	GGGATAGCAGAGAGTAAATTGG
	Dpu_11130R2	CTTCTACAGGCTTTGCTCCGATTC
	Dpu_11445R2	TAGGTTAAACAATCTTATGC
	Dpu_12035R2	TTGGATGTCTACGAGGAGTAGCCCA
	Dpu_12336R2	TACATTCAGTTGCGTAAGGGGC
	Dpu_12606R2	AGCCAGGTTGGTTTCTATCCTC
	Dpu_12761R2	CTAGGGATAACAGCGTAATC
	Dpu_13323R2	GTGTTTATCTATCTACCAGAATAC
	Dpu_13787R2	CCATCTTTGTCCAAATTGTG
	**Dpu_14188R2**	AGGCTTAAAACTCAGGTGAAGGTGG
Set 3	**Dpu_13944F3**	TTGAGAAGAGAGTGACGGGCGATAT
	Dpu_14626F3	AGCCAGATTCAAACTTTCCC
	Dpu_14685F3	CGGAACCACTTTAGCGCAAGTA
	Dpu_00747R3	CTGTCATCAACCAGCCTACATG
	Dpu_01182R3	GAAAGTAGATCATGGATAGC
	Dpu_01385R3	ATGGCCGAGGAGTAGGCATTAA
	**Dpu_02003R3**	TGCTAGGACTGGTAGGCTCAATAG
